# Effect of exercise training on nitric oxide and nitrate/nitrite (NOx) production: A systematic review and meta-analysis

**DOI:** 10.3389/fphys.2022.953912

**Published:** 2022-10-04

**Authors:** Tahereh Arefirad, Ehsan Seif, Mahdi Sepidarkish, Nami Mohammadian Khonsari, Seyedeh Azam Mousavifar, Shahrooz Yazdani, Fatemeh Rahimi, Faezeh Einollahi, Javad Heshmati, Mostafa Qorbani

**Affiliations:** ^1^ Department of Exercise Physiology, Science and Research Branch, Islamic Azad University, Tehran, Iran; ^2^ Non-communicable Diseases Research Center, Alborz University of Medical Sciences, Karaj, Iran; ^3^ Department of Biostatistics and Epidemiology, School of Public Health, Babol University of Medical Sciences, Babol, Iran; ^4^ Social Determinants of Health Research Center, Alborz University of Medical Sciences, Karaj, Iran; ^5^ Department of Health Education and Health Promotion, Faculty of Health, Isfahan University of Medical Sciences, Isfahan, Iran; ^6^ Cardiovascular Research Center, Shahid Rajaei Educational & Medical Center, Alborz University of Medical Sciences, Karaj, Iran; ^7^ Songhor Healthcare Center, Kermanshah University of Medical Sciences, Kermanshah, Iran; ^8^ Endocrinology and Metabolism Research Center, Endocrinology and Metabolism Clinical Sciences Institute, Tehran University of Medical Sciences, Tehran, Iran

**Keywords:** exercise, training, aerobic, nitric oxide, NO

## Abstract

**Background:** Exercise and physical activity can improve circulation through various mechanisms, such as the increment of nitric oxide (NO) production, by affecting vascular endothelial nitric oxide synthase, and reducing reactive oxygen species (ROS). Although, theoretically, this mechanism is well known, studies in living subjects have made controversial findings regarding the association of NO production and its metabolites [nitrate/nitrite (NOx)] with physical activity. Hence, this systematic review and meta-analysis was designed to gather all these studies and evaluate the effects of exercise training, and physical activity duration and length on the mean change of serum/plasma NO and NOx.

**Method:** We searched all available bibliographic electronic databases from inception through to May 2022 to include all randomized controlled trials (RCT) and quasi-experimental trials which assessed the effect of exercise and training on NO and NOx levels. Random-effects meta-analysis was used to pool the standardized mean difference (SMD) and 95% confidence interval (CI) of included RCT studies which assessed the effect of training. Stratified meta-analysis was performed according to the type of exercise (high-intensity interval training (HIIT), aerobic training (AT), the duration of exercise (≤8 and > 8 weeks), and length of exercise in each session ≥40 and 40 < minutes).

**Results:** Overall, 15 and 10 studies were included in the systematic review and meta-analysis, respectively. According to the random-effects meta-analysis, exercise significantly increased the mean change of NO and NOx compared to control (SMD: 1.82, 95%CI: 1.14 to 2.49. In the stratified meta-analysis, the mean change of NO and NOx in the intervention group was significantly higher than in the control group in the AT (SMD: 1.36, 95%CI: 0.55–2.18), HIIT (SMD: 2.55, 95%CI: 1.14–3.96), duration of ≤8 (SMD: 2.29, 95%CI: 1.24–3.35) and > 8 weeks (SMD: 1.19, 95%CI: 0.52–1.86), length of ≥40 (SMD: 1.61, 95%CI: 1.04–2.18), and 40 < minutes in each session (SMD: 2.07, 95%CI: 0.79–3.35).

**Conclusion:** The findings of this study indicate that, regardless of exercise duration, length, and type (AT or HIIT), exercise can significantly increase serum NO and NOx levels.

## Background

Cardiovascular disease (CVD) is one of the major causes of disability and mortality in both developing and developed communities (Balakumar et al., 2016). The health of vascular endothelium tissue plays a major role in the incidence and control of cardiovascular diseases (Quyyumi, 1998). Vascular endothelium, a layer on the internal area of vessels, can produce various substances that control vascular constriction and relaxation (Förstermann et al., 2017). One of these substances is nitric oxide (NO)—later metabolized to nitrate/nitrite (NOx)—a cell-signaling molecule that is produced in endothelium through an enzymatic transformation of l-arginine by NO synthase (NOS) (Li et al., 2014). NOS presents in many cell types and tissues, including blood vessels, nerve cells, smooth muscles, myocytes, macrophages, and kidney endothelial cells (Di Pietro et al., 2017). The release of NO by relaxation of the vascular smooth muscles in the systemic and cerebral circulation plays a vital role in preventing CVD (Bondonno et al., 2016). The positive influences of physical activity and exercise on CVD control are well established (Bohn et al., 2015). Exercise-induced improvement of endothelial function can lead to a decrease in cardiovascular complications (Linke et al., 2006). Regular aerobic training (AT) and mild to moderate exercise are firmly prescribed for people who are not physically active, due to their beneficial impact on glycemic control and insulin sensitivity (Boulé et al., 2001). It has also been demonstrated that people with diabetes and CVD will benefit from such training and workouts as these have beneficial effects on lipid and glycemic control, postprandial glycemia and lipemia, fasting blood lipids, body mass, and blood pressure (Duncan, 2006; Harding et al., 2006).

Several pieces of research have demonstrated that AT can improve endothelium-dependent vasodilation by increasing NOS expression and phosphorylation (Linke et al., 2006; Gómez-Guzmán et al., 2012; Dyakova et al., 2015). Recent studies have also shown that exercise increases NO and, consequently, NOx production in the body (Shen et al., 1995). Another study on animals and humans has indicated that obesity reduces the bioavailability of NO (Lemaster et al., 2016). Even though several studies have examined the effect of different types of exercise training on NO and NOx production and, although theoretically, this mechanism is well known (Nosarev et al., 2014; Dyakova et al., 2015), the association between different modalities, lengths, and durations of physical activity, and NO/NOx production among studies in living subjects, are controversial (Nosarev et al., 2014). Moreover, no systematic review and meta-analysis has pooled the effect of exercise training on NO and NOx production. Therefore, this systematic review and meta-analytical study was designed to evaluate and pool the effect of different exercise durations, lengths, and modalities on the levels of serum/plasma NO and NOx.

## Methods

### Search strategy

A global systematic review of electronic databases was conducted to identify controlled clinical trials that have evaluated the effect of physical activity on circulating concentrations of serum/plasma NO and NOx. The search followed the guidelines of the 2009 preferred reporting items for systematic reviews and meta-analysis (PRISMA) statement. MEDLINE, Web of Sciences, EMBASE, Scopus, and the Cochrane Central Register of Controlled Trials (CENTRAL) electronic databases were searched up to May 2022 with search terms “NO” combined with exercise training and physical activity. The entire search details are available in Supplementary Appendix S1. In addition, reference lists of the relevant reviews and included studies were investigated to identify other potentially eligible articles. No language and publication date restrictions were used in the literature search. Two independent researchers (JH and ES) evaluated the retrieved articles by screening the title and abstract, then by a full-text evaluation. Disagreements regarding study selection were resolved through discussion.

### Criteria for considering studies for this review

Studies were considered eligible for inclusion in this review if they met the following criteria (Balakumar et al., 2016): being a randomized controlled trial (RCT) with parallel design or quasi-experimental trial (before–after) (Quyyumi, 1998), an intervention study that applied physical activity as monotherapy or as adjunctive therapy provided that appropriate control group is included (Förstermann et al., 2017), reporting enough data on NO parameters at the beginning and that the end of the study in intervention and control groups (Li et al., 2014), had evaluated serum/plasma concentration of NOx. Exclusion criteria included (Balakumar et al., 2016): observational studies (cohort, case studies, case series, case–control, and cross-sectional) (Quyyumi, 1998), studies that did not report enough data on methodology or results (Förstermann et al., 2017), and studies that had evaluated exhaled or urinary excretion of NOx. Furthermore, the systematic review included all RCTs and quasi-experimental trials on NOx. However, only those studies with two parallel group designs as intervention and control groups—where the control group was sedentary without any exercise—were included in the meta-analysis. Moreover, if a study had assessed the effect of different exercise modes on NO and NOx in comparison with the control group, each comparison was considered a separate study in the meta-analysis.

### Data extraction and quality assessment

The following data of eligible studies were extracted by the two reviewers (JH and ES) independently using a standard protocol. The extracted data included (Balakumar et al., 2016) the first author’s name (Quyyumi, 1998), publication date (Förstermann et al., 2017), study location (Li et al., 2014), number of participants in the case and control groups (Di Pietro et al., 2017), study design (Bondonno et al., 2016), information regarding randomization, blinding and drop-outs (Bohn et al., 2015), duration of intervention (Linke et al., 2006), means, and standard deviations of serum/plasma NO and NOx in treatment and control groups.

Eligible trials were methodologically and separately evaluated for their quality using the Cochrane risk of bias tool. This tool evaluates six parameters: selection bias (method for random sequence generation and allocation concealment), performance bias (blinding of participants and personnel), detection bias (blinding of outcome assessment), attrition bias (incomplete outcome data), reporting bias (selective reporting), and other sources of bias.

### Ethical considerations

This current systematic review was approved by the ethical committee of the Alborz University of Medical Sciences. All primary articles included were cited in all reports and in the final manuscript.

### Statistical analysis

Meta-analysis was conducted using Stata 17.0 software (Stata Corp, College Station, Texas). The result of each study was presented as a standardized mean difference (SMD) and 95% confidence interval (CI). Heterogeneity between the included studies was assessed using the Q-Cochrane test and was regarded as statistically significant at *p* < 0.1. The degree of heterogeneity was estimated using I^2^ statistics. In cases of significant heterogeneity between studies, the pooled SMD as a measure of effect size was estimated using the random-effects model (Der-Simonian and Laird method); otherwise, a fixed-effect model was used. Publication bias was scrutinized using Egger’s test. A *p*-value <0.10 was considered a statistically significant publication bias. If publication bias was significant, the trim and fill correction was used to impute missing studies and correct and adjust the pooled SMD. To determine each study’s effect on the overall pooled association of serum/plasma NO and NOx concentration with exercise, sensitivity (leave-one-out) analysis was also performed. Moreover, according to previous meta-analyses, a stratified meta-analysis was performed according to the type of exercise modes (high-intensity interval training (HIIT) and AT), length of exercise (≤8 weeks/> 8 weeks), and its duration (<40 min per session/≥40 min per session) (Wenger and Bell, 1986; Clark, 2016). All comparisons were two-tailed, with 95% CIs described if applicable.

## Results

### Literature search

**FIGURE 1:**
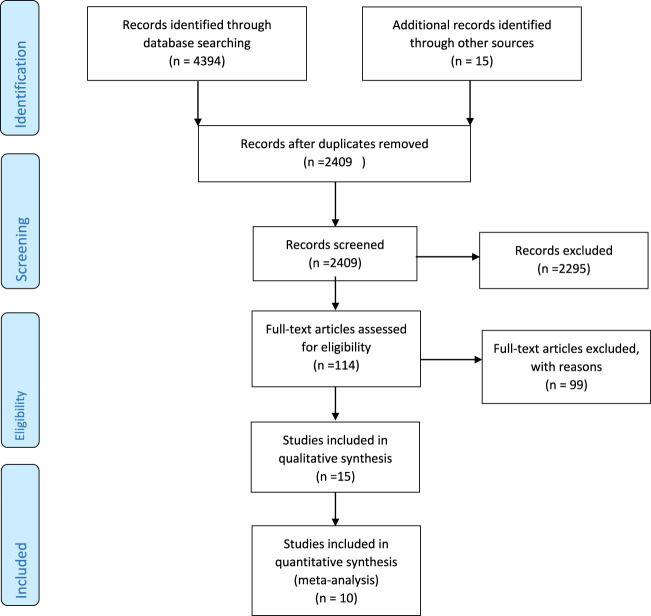
PRISMA flow diagram of included studies.

The primary database search identified 4394 relevant articles. There were 2409 unique articles after removing duplicates, and 2295 were further excluded due to irrelevant content. Of the 114 full-text screened articles, 104 did not qualify for inclusion criteria either because they lacked sufficient information on the outcomes of interest, inappropriate design, or did not respond to our email request for additional data. In the end, 15 and 10 studies were included in the qualitative synthesis and meta-analysis, respectively, with each study containing two different interventions and one control group (Figure 1).

### Summary of study characteristics and main findings

The main characteristics and findings of all 15 included studies (Higashi et al., 1999; Narin et al., 2003; Maeda et al., 2004; Wang, 2005; Mourot et al., 2009; Krause et al., 2014; Elsisi et al., 2016; Ghardashi Afousi et al., 2016; Tomeleri et al., 2017; Tsukiyama et al., 2017; Ghahremani Moghadam and Hejazi, 2018; Hasegawa et al., 2018; Izadi et al., 2018; Mohammadi et al., 2018; Arefirad et al., 2020) in the systematic review are presented in Table 1 comprising 527 participants. Ten studies were included in the meta-analysis, comprising 265 participants (Higashi et al., 1999; Narin et al., 2003; Maeda et al., 2004; Ghardashi Afousi et al., 2016; Tomeleri et al., 2017; Ghahremani Moghadam and Hejazi, 2018; Hasegawa et al., 2018; Izadi et al., 2018; Mohammadi et al., 2018; Arefirad et al., 2020). All studies were performed from 1999 to 2020, with sample sizes ranging from 12 to 60 healthy or unhealthy individuals. Intervention durations ranged from three to 16 weeks. The age of participants ranged from 20 to 70 years. Overall, 13 studies were RCTs and two were quasi-experimental. The majority of the studies were conducted in Iran (five), followed by Japan (four), and Ireland, Egypt, France, Turkey, Taiwan, and Brazil (one study each). Based on exercise training mode, there were five studies on AT, five on HIIT, and one on resistance training. Of these, three found a significant association between serum/plasma NOx levels and AT, and five found a significant association between serum/plasma NOx levels and HITT.

**TABLE 1:** General characteristics of included studies.

Author, year (References)	Country	Study design	Participants	Sample size (Total)	Exercise characteristics			Main findings
					Type	Intensity (%VO_2_ex/VO_2_max; %HRex/HRmax)	Duration (weeks); frequency (per week); length in each session (minutes)	
Ghahremani-moghadam et al., 2018 (Ghahremani Moghadam and Hejazi, 2018)	Iran	RCT	Sedentary elderly women	21	AT	50–70 (%HRex/HRmax)	8; 3; 45–60	↑NO, ↓8-hydroxydeoxyguanosine
Izadi et al., 2017 (Izadi et al., 2018)	Iran	RCT	Hypertensive subjects	30	HIIT	85–90 (%HRex/HRmax)	6; 3; 35	↓ET-1, ↑apelin, ↑NOx
Krause et al., 2013 (Krause et al., 2014)	Ireland	RCT	Obese men suffering from DM vs. obese healthy	12	AT (FatMax)	30–40 (%VO_2_ex/VO_2_max)	16; 3; 30	Baseline serum tNOx ↑ in controls than T2DM, ↔nNOS and tNOx
			Obese men suffering from DM vs. obese healthy	13	AT (Tvent)	55–65 (%VO_2_ex/VO_2_max)	16; 3; 30	Baseline serum tNOx ↑ in controls than T2DM, ↑nNOS and tNOx in the control group only
Mohammadi et al., 2018 (Mohammadi et al., 2018)	Iran	RCT	Overweight elderly men	24	HIIT	90 (%HRex/HRmax)	8; 3; 40–60	↑NO, ↓ ET-1
Narin et al., 2003 (Narin et al., 2003)	Turkey	RCT	Women with general migraine	40	AT	(-)	8; 3; 60	↑NO
Hasegawa et al., 2018 (Hasegawa et al., 2018)	Japan	RCT	Healthy volunteers	21	Control	40 (%VO_2_ex/VO_2_max); ≥90 (%HRex/HRmax) for AT; 170 (%VO_2_ex/VO_2_max); ≥90 (%HRex/HRmax) for HIIT	6; 4; 20 for HIIT—8; 4; 20 for AT	↑NOx in both AT and HIIT groups compared with the control group
					AT			
					HIIT			
Higashi et al., 1999 (Higashi et al., 1999)	Japan	RCT	HTN patients	17	AT	52 ± 9 (%VO_2_ex/VO_2_max)	12; 6; 30	↑NOx, ↑response to acetylcholine
Mourot et al., 2009 (Mourot et al., 2009)	France	RCT	Men suffering from stable CAD or CHF	24 each (CAD and CHF)	AT plus gymnastic exercise on land	60–70 (%VO_2_ex/VO_2_max)	3; 5; 30 min aerobic plus 50 min gymnastics	↔ NOx, ↔catecholamine
			Men suffering from stable CAD or CHF	24 each (CAD and CHF)	AT plus gymnastic exercise in water	(-)	3; 5; 30 min aerobic plus 50 min gymnastics	↑nitrate, ↔nitrite, ↔catecholamine
Maeda et al., 2004 (Maeda et al., 2004)	Japan	RCT	Elderly women	15	AT	50 (%VO_2_ex/VO_2_max); 80 (%HRex/HRmax)	13; 5; 30	↑NOx, ↑cGMP
Tomeleri et al., 2016 (Tomeleri et al., 2017)	Brazil	RCT	Elderly women suffering from HTN	30	RT	(-)	12; 2; 10	↑NOx
Wang et al., 2004 (Wang, 2005)	Taiwan	Quasi-experimental	Healthy adult	20	AT	50 (%VO_2_ex/VO_2_max)	8; 5; 30	↑NOx
Tsukiyama et al., 2017 (Tsukiyama et al., 2017)	Japan	Quasi-experimental	Healthy adult (including some trained adults)	40	AT	60 (%HRex/HRmax)	4; 5; 60	↔NOx, ↑NO2
Ghardashi Afousi et al., 2016 (Ghardashi Afousi et al., 2016)	Iran	RCT	Patients suffering from DM and HTN	30	HIIT	(-)	10; 3; 40	↑NOx, ↔flow-mediated dilation
Elsisi et al., 2016 (Elsisi et al., 2016)	Egypt	RCT	Women suffering from DM2	60	AT vs. HIIT	(-)	8; 3; 25	↑NO, ↑NO significantly more in HIIT
Arefirad et al., 2019 (Arefirad et al., 2020)	Iran	RCT	Women suffering from DM	30	HIIT	90 (%HRex/HRmax)	6; 3; 25	↑NOx

HIIT: high-intensity interval training; AT: aerobic training; NO: nitric oxide; NOx: nitrite/nitrate; nNOS: nitric oxide synthase; ET: endothelin; PWV: pulse wave velocity; cGMP: cyclic guanosine monophosphate; BP: blood pressure; HR: heart rate; Ach: acetylcholine; DM: diabetes mellitus; ex: during exercise; max: maximum; VO2: oxygen consumption*; ↓ This symbol is a sign of decreasing variables in the intervention group; ↑ This symbol is a sign of increasing variables in the intervention group; ↔ This sign indicates that there is no difference between the two groups; NR: not reported.

**FIGURE 2:**
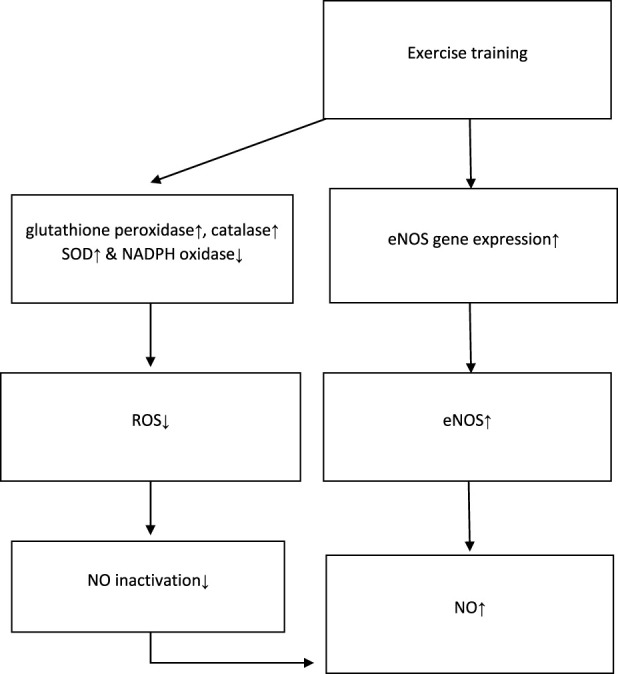
Possible mechanisms of NO increment proposed by studies. NO: nitric oxide; eNOS: endothelial nitric oxide synthase; ROS: reactive oxygen species; SOD: superoxide dismutase; NADPH: nicotinamide adenine dinucleotide phosphate. ↓ This symbol is a sign of decreasing variables in the intervention group. ↑ This symbol is a sign of increasing variables in the intervention group.

The possible mechanisms, proposed by the majority of studies, are presented in Figure 2. According to this figure, exercise training increases the serum/plasma NOx levels either *via* the increment of eNOS or reduction of NO clearance.

### Risk of bias assessment

Supplementary Appendix S2 provides the methodological features of included trials in the meta-analysis. All assessed studies were considered as being at high risk of bias for allocation concealment and blinding participants and personnel because detailed blinding processes were not found in any of the included RCTs.

### Serum/plasma NOx

**FIGURE 3:**
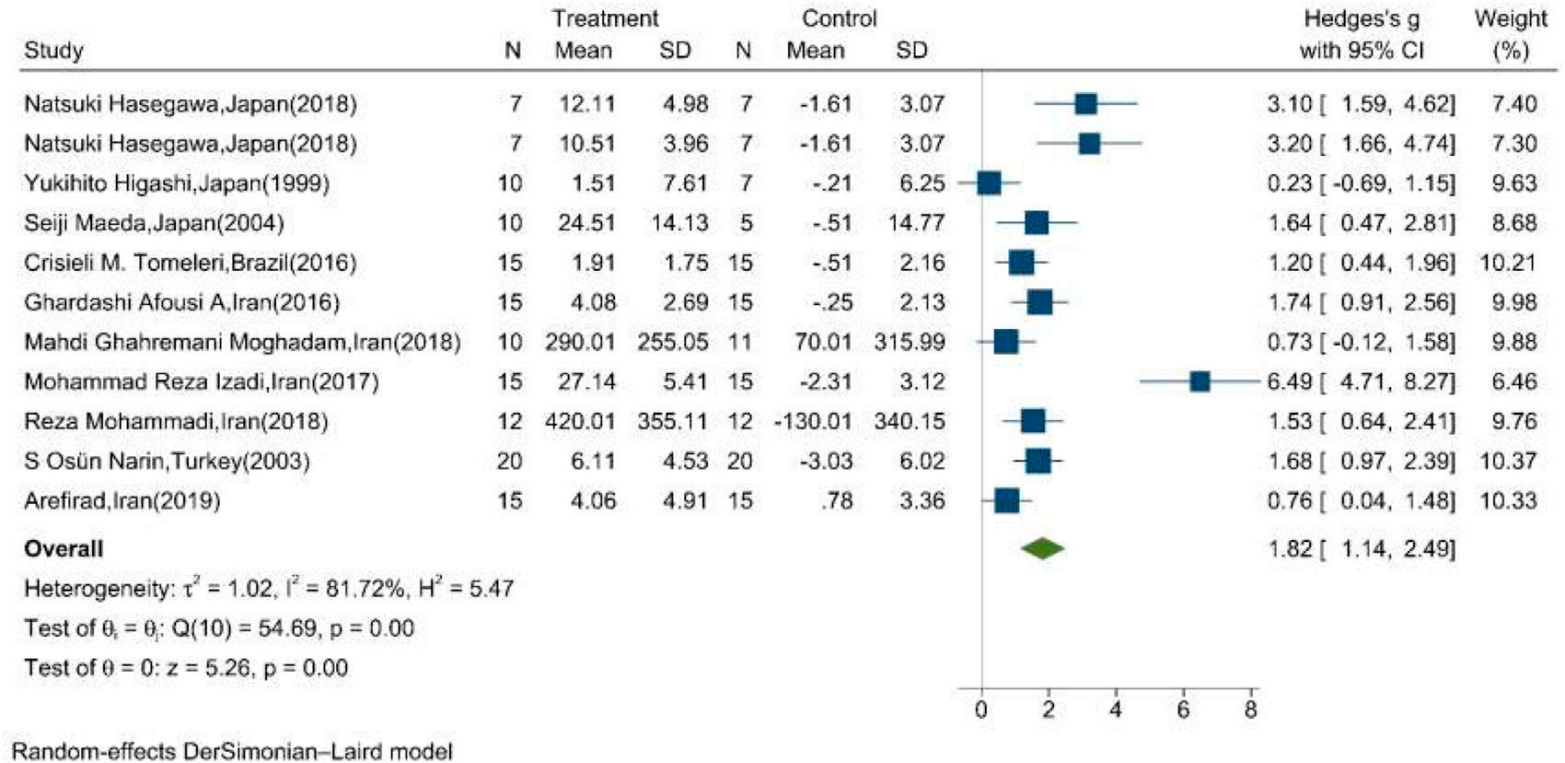
Forest plot for the effect of training on mean change of serum NOx concentration. (The vertical line represents null effect.)

The mean changes of serum/plasma NOx as the final metabolites of produced NO were assessed between the exercise and control groups in ten articles, in which one study performed two exercise training modes in comparison with the control groups (Hasegawa et al., 2018). The random effect meta-analysis model (Q = 54.69, *p*-value < 0.01, I^2^ = 81.72%) showed a significant association between exercise training and serum/plasma NOx with a pooled SMD of 1.82 (95%CI: 1.14–2.49) (Figure 3).

### Publication bias

The results of Egger’s test supported the existence of publication bias regarding the effect of exercise training on serum/plasma NOx levels (β: 7.31, SE: 1.46, *p*-value<0.001). Nonetheless, upon “trim and fill” correction, no missing studies were imputed and the results of the analysis did not change; therefore, publication bias did not substantially affect the results.

### Stratified meta-analysis

**FIGURE 4:**
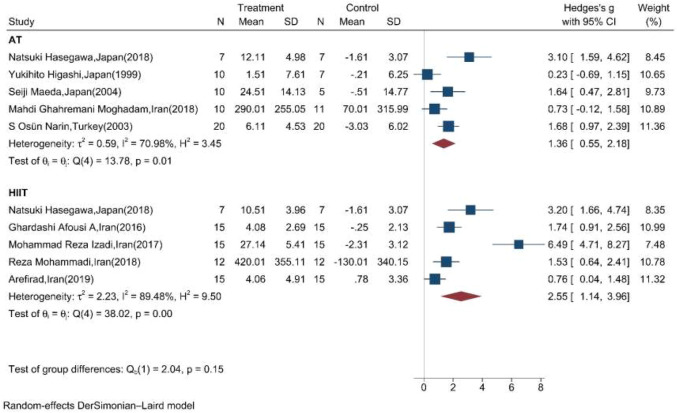
Forest plot for the effect of training on mean change of serum NOx concentration according to the type of training (AT: aerobic training; HIIT: high-intensity interval training. The vertical line represents null effect).

Table 2 shows the results of the subgroup meta-analysis according to the type of exercise training mode, duration of exercise in terms of weeks, and length of each session. Random effect sub-group meta-analysis showed that the effects of HIIT and AT on the mean change of serum/plasma NOx concentration were statistically significant with severe heterogeneity between studies (Q = 38, *p* < 0.01, I^2^ = 89.5%) (pooled SMD: 2.55, 95%CI: 1.14–3.96) and (Q = 14.8, *p* = 0.01, I^2^ = 71%) (pooled SMD: 1.36, 95%CI: 0.55–2.18), respectively (Figure 4).

**TABLE 2:** Pooled effect of exercise training on nitric oxide level in meta-analysis.

Subgroup	Sample size	SMD (95% CI)	Model	Heterogeneity assessment		
				I^2^%	Q test	*p*-Value of heterogeneity
Overall	265	1.82 (1.14_2.49)^b^	Random-effects	81.7	54.7	<0.01
By exercise type^a^						
AT	107	1.36 (0.55_2.18)^b^	Random-effects	71.0	14.8	0.01
HIIT	128	2.55 (1.14_3.96)^b^	Random-effects	89.5	38.0	<0.01
By duration of intervention						
≤8 weeks	173	2.29 (1.24 _3.35)^b^	Random-effects	89.9	60.0	<0.01
>8 weeks	92	1.19 (0.52_1.86)^b^	Random-effects	52.48	7.5	0.06
By duration of exercise per session						
<40 min	136	2.07 (0.79_3.35)^b^	Random-effects	89.2	46.2	<0.01
≥40 min	129	1.61 (1.04_2.18)^b^	Random-effects	49.7	7.9	0.09

a Resistance training method dropped off since there was only one study.

SMD: standardized mean difference; AT: aerobic training; HIIT: high-intensity interval training; min: minutes.

b Statistically significant.

**FIGURE 5:**
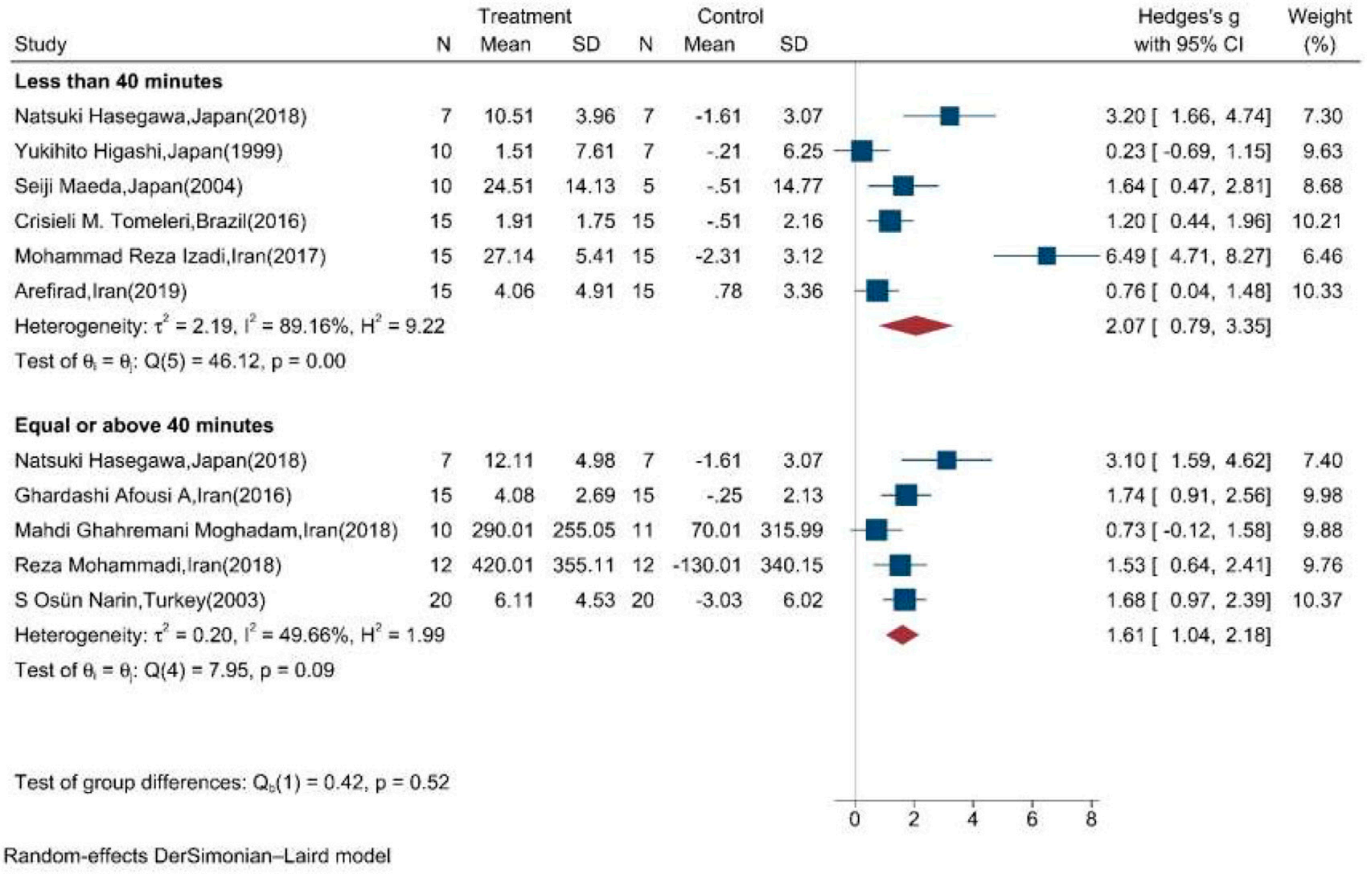
Forest plot for the effect of training on mean change of serum NOx according to the length of training in each session. (The vertical line represents null effect.)

Subgroup meta-analysis according to the length of exercise training in each session (<40 min per session/≥40 min per session) showed that both types of length significantly increased the mean change of NOx in the intervention compared to the control group (Figure 5).

**FIGURE 6:**
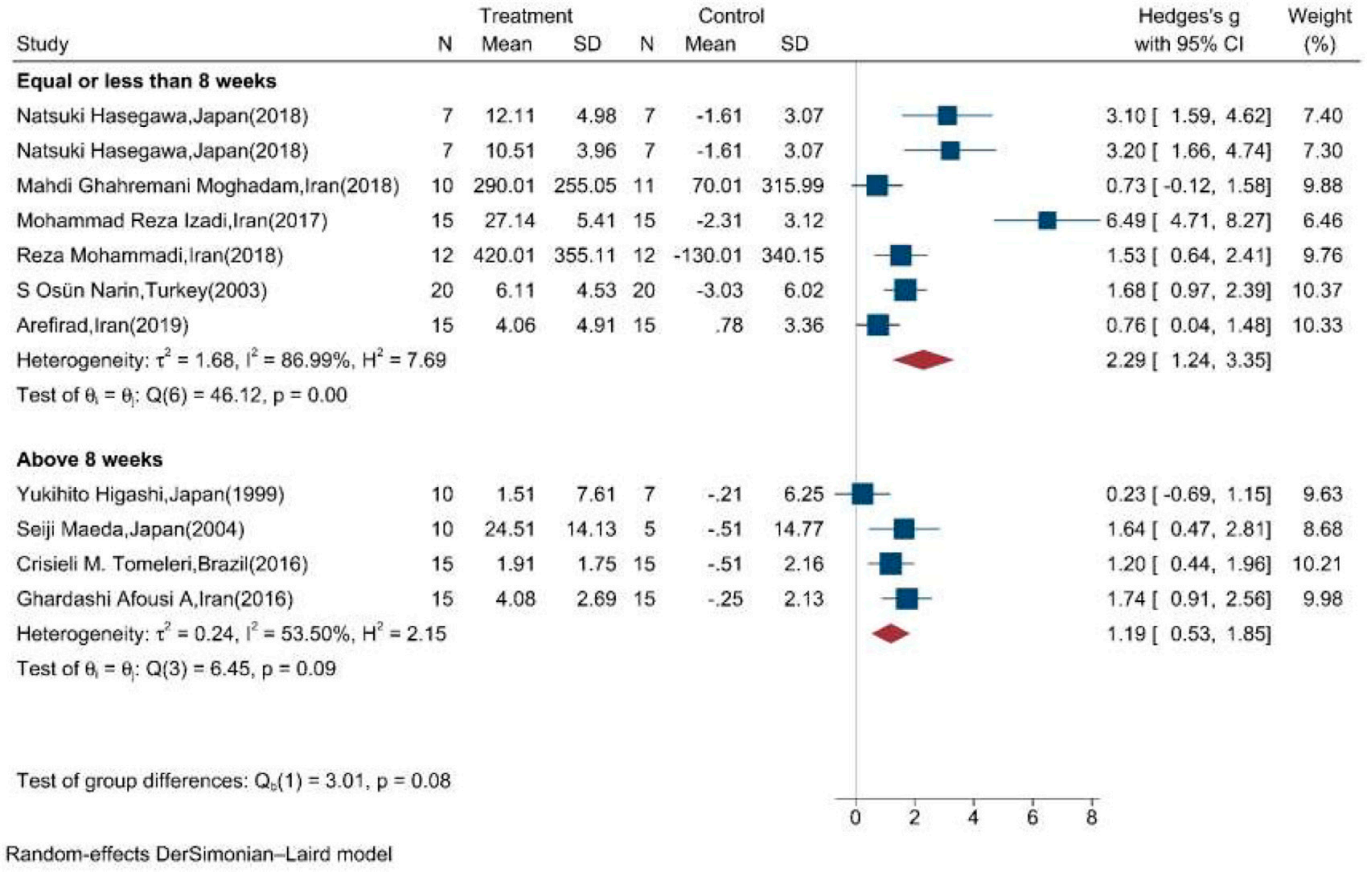
Forest plot for the effect of training on mean change of serum NOx concentration according to the duration of training initiation. (The vertical line represents null effect.)

Moreover, the random effect sub-group meta-analysis showed that the duration of exercise training equal, less, and above eight weeks significantly increased the mean concentration of NOx (Figure 6).

### Sensitivity analysis

**FIGURE 7:**
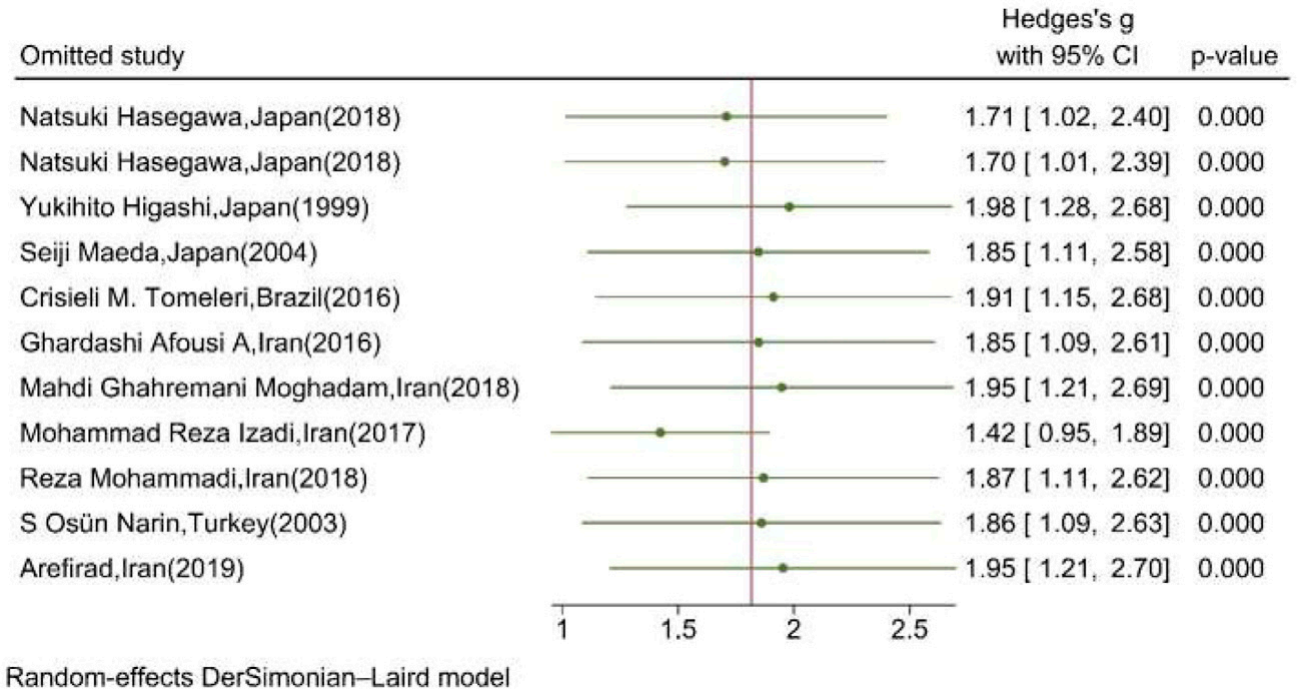
Leave-one-out analysis illustrating the overall pooled association of serum/plasma NOx levels upon omitting each study. (The vertical line represents the overall effect of training on the mean change of serum NOx.)

Figure 7 shows the results of the “leave-one-out” analysis. Upon omitting each study, the overall pooled association of serum/plasma NOx levels and exercise remained significant, meaning that the significance of the association was not substantially affected by the results of each study.

## Discussion

To the best of our knowledge, this is the first systematic review and meta-analysis that has assessed and pooled the effect of exercise training on serum/plasma NO and NOx levels. In our meta-analysis, exercise significantly increases serum/plasma NOx levels. These results accord with those of Olszanecka-Glinianowicz et al. (2008), Bechara et al. (2008), and Chikara Goto et al. (2003).

In the studies included, some mechanisms have been suggested to explain the association between exercise and serum/plasma NO and NOx levels. Previous studies have shown that exercise increases eNOS gene expression in the lungs, increasing nitric acid production from arginine (Miyauchi et al., 2003). It is also established that aerobic exercise training increases eNOS genes, phosphorylation, and NO levels in the aorta (Franklin et al., 2020).

As previously mentioned (Figure 2), most studies propose that physical activity stimulates the eNOS which lies near the sarcolemma and dystrophin complex within the muscle fibers, thus increasing NO production (Tidball and Wehling-Henricks, 2014). Furthermore, another significant mechanism discussed by studies was that reduced NO bioavailability resulted from its direct quenching by superoxide, even without altering NOS activity (Paolocci et al., 2001). In this regard, it has been demonstrated that regular exercise improves endothelial function by increasing vascular endothelial growth factor-induced angiogenesis and reduces NO deactivation with the reinforcement of components of the antioxidant defense system functions, such as glutathione peroxidase, catalase, and superoxide dismutase (SOD). It decreases nicotinamide adenine dinucleotide phosphate (NADPH) oxidase-derived production of reactive oxygen species (ROS) and angiotensin receptor I and II expression, leading to elevated NO bioavailability and vasodilation (Hollander et al., 1999; Linke et al., 2006). These studies suggest that training and exercise increase NO generation and reduce NO deactivation, thus increasing serum/plasma NO levels (Powers et al., 2016).

The stratified meta-analysis of our findings showed that the effect of exercise on NOx on both AT and HIIT, duration of exercise ≤8 and >8 weeks, and duration of exercise ≥40 and <40 min in each session were statistically significant, thus promoting NO production. Regular exercise can improve body composition and cardiovascular efficiency (Gibson et al., 2018). NO also plays an essential role in the control of CVD by improving endothelial function and vasodilation, glucose homeostasis, and insulin resistance (Lundberg et al., 2015). However, the mechanism associated with training-induced eNOS activation is still unclear; however, mechanical pressures such as shear stress, known to elevate during AT, have been recognized as a major stimulus for eNOS activation (Boo et al., 2002; Tang et al., 2006). Therefore, the intensity of exercise in studies can vary, which may lead our study to differ from the outcomes of previous studies. Moreover, further research is needed to elucidate the actual effect of physical activity on NO levels in those with comorbid disease.

## Limitations and strength

This systematic review and meta-analysis faced some limitations. First, the included studies were heterogeneous in regard to training mode, duration of intervention, and length. Second, the heterogeneity between studies in terms of the study population (healthy, subjects with metabolic disorders, and subjects with hypertension) in this review limited our findings. Moreover, the variation in control groups in included studies made it difficult to precisely conclude that the pooled estimated effect is attributed to the training effect. In addition, although the sample size was small in most of the included studies and, in the meta-analysis, the sample and the effect size were pooled, the pooled sample size was not large enough, particularly in the subgroup analysis (according to training mode, prolonged/shortened training time in each session, and duration of exercise training) to robustly justify the findings. Nonetheless, the present systematic review and meta-analysis were based on a comprehensive literature search; it is thus unlikely that much related research was overlooked.

## Conclusion

The findings of this study indicate that, regardless of duration, length, and type (AT or HIIT), exercise can significantly increase serum NOx levels. Further randomized trials in different study populations (healthy/unhealthy) with larger sample sizes, homogenous duration, and similar training are needed to approve the findings of this systematic and meta-analytical review.

## Data Availability

The original contributions presented in the study are included in the article/Supplementary Material; further inquiries can be directed to the corresponding authors.
