# Supplementary material for: Effect of exercise training on nitric oxide and nitrate/nitrite (NOx) production: A systematic review and meta-analysis
Source: Front Physiol. 2022 Oct 4;13:953912. doi: 10.3389/fphys.2022.953912 (PMC9576949; doi:10.3389/fphys.2022.953912)
Supplement: Supplementary file 1 [file Table1.DOCX]

**Appendix 1: Search strategy**

| **Groups** | **Descriptors** |
| --- | --- |
| Outcome | Nitric Oxide OR Nitrogen Monoxide OR Nitrogen oxide OR Nitric Oxide, Endothelium-Derived OR Endogenous Nitrate Vasodilator OR Nitrates OR nitrite OR Nitrosamines OR Nitrogen dioxide |
| Intervention | Exercise OR Physical Activity OR Training, Exercise OR Sport OR aerobic training OR anaerobic training OR endurance training OR resistance  training OR interval training OR walking OR muscle strengthening |
| Setting | Randomized controlled trial OR controlled clinical trial OR randomized controlled trials OR random allocation OR double blind method OR single blind method OR clinical trial OR clinical trials OR placebos OR placebo OR random |

**PUBMED**

|  | **Descriptors** |
| --- | --- |
| **#1** | ((((((((("nitric oxide"[MeSH Terms] OR ("nitric"[All Fields] AND "oxide"[All Fields]) OR "nitric oxide"[All Fields]) OR ("nitric oxide"[MeSH Terms] OR ("nitric"[All Fields] AND "oxide"[All Fields]) OR "nitric oxide"[All Fields] OR ("nitrogen"[All Fields] AND "monoxide"[All Fields]) OR "nitrogen monoxide"[All Fields])) OR ("nitrogen oxides"[MeSH Terms] OR ("nitrogen"[All Fields] AND "oxides"[All Fields]) OR "nitrogen oxides"[All Fields] OR ("nitrogen"[All Fields] AND "oxide"[All Fields]) OR "nitrogen oxide"[All Fields])) OR ("nitric oxide"[MeSH Terms] OR ("nitric"[All Fields] AND "oxide"[All Fields]) OR "nitric oxide"[All Fields] OR ("nitric"[All Fields] AND "oxide"[All Fields] AND "endothelium"[All Fields] AND "derived"[All Fields]) OR "nitric oxide, endothelium derived"[All Fields])) OR ("nitric oxide"[MeSH Terms] OR ("nitric"[All Fields] AND "oxide"[All Fields]) OR "nitric oxide"[All Fields] OR ("endogenous"[All Fields] AND "nitrate"[All Fields] AND "vasodilator"[All Fields]) OR "endogenous nitrate vasodilator"[All Fields])) OR ("nitrates"[MeSH Terms] OR "nitrates"[All Fields])) OR ("nitrites"[MeSH Terms] OR "nitrites"[All Fields] OR "nitrite"[All Fields])) OR ("nitrosamines"[MeSH Terms] OR "nitrosamines"[All Fields])) OR ("nitrogen dioxide"[MeSH Terms] OR ("nitrogen"[All Fields] AND "dioxide"[All Fields]) OR "nitrogen dioxide"[All Fields])) OR ("nitric oxide"[MeSH Terms] OR ("nitric"[All Fields] AND "oxide"[All Fields]) OR "nitric oxide"[All Fields] OR "no"[All Fields]) |
| **#2** | (((((((((("exercise"[MeSH Terms] OR "exercise"[All Fields]) OR ("exercise"[MeSH Terms] OR "exercise"[All Fields] OR ("physical"[All Fields] AND "activity"[All Fields]) OR "physical activity"[All Fields])) OR ("exercise"[MeSH Terms] OR "exercise"[All Fields] OR ("training"[All Fields] AND "exercise"[All Fields]) OR "training, exercise"[All Fields])) OR ("sports"[MeSH Terms] OR "sports"[All Fields] OR "sport"[All Fields])) OR (aerobic[All Fields] AND ("education"[Subheading] OR "education"[All Fields] OR "training"[All Fields] OR "education"[MeSH Terms] OR "training"[All Fields]))) OR (anaerobic[All Fields] AND ("education"[Subheading] OR "education"[All Fields] OR "training"[All Fields] OR "education"[MeSH Terms] OR "training"[All Fields]))) OR (endurance[All Fields] AND ("education"[Subheading] OR "education"[All Fields] OR "training"[All Fields] OR "education"[MeSH Terms] OR "training"[All Fields]))) OR ("resistance training"[MeSH Terms] OR ("resistance"[All Fields] AND "training"[All Fields]) OR "resistance training"[All Fields])) OR (interval[All Fields] AND ("education"[Subheading] OR "education"[All Fields] OR "training"[All Fields] OR "education"[MeSH Terms] OR "training"[All Fields]))) OR ("walking"[MeSH Terms] OR "walking"[All Fields])) OR (("muscles"[MeSH Terms] OR "muscles"[All Fields] OR "muscle"[All Fields]) AND strengthening[All Fields]) |
| **#3** | ((((((((("Randomized Controlled Trial"[Publication Type] OR "Controlled Clinical Trial"[Publication Type]) OR "Randomized Controlled Trials as Topic"[Mesh]) OR "Random Allocation"[Mesh]) OR "Double-Blind Method"[Mesh]) OR "Single-Blind Method"[Mesh]) OR "Clinical Trial"[Publication Type]) OR ("clinical trial"[Publication Type] OR "clinical trials as topic"[MeSH Terms] OR "clinical trials"[All Fields])) OR "Placebos"[Mesh]) OR ("placebos"[MeSH Terms] OR "placebos"[All Fields] OR "placebo"[All Fields])) OR ("random allocation"[MeSH Terms] OR ("random"[All Fields] AND "allocation"[All Fields]) OR "random allocation"[All Fields] OR "random"[All Fields]) |
| **#4** | **#1** AND **#2** AND #3 |

**WEB OF SCIENCE**

|  | **Descriptors** |
| --- | --- |
| **#1** | TS=(“Exercise ”) OR TS=(“Physical Activity”) OR TS=(“Training, Exercise”) OR TS=(“Sport”) OR TS=(“aerobic training”) OR TS=(“anaerobic training”) OR TS=(“endurance training”) OR TS=(“resistance training”) OR TS=(“interval training”) OR TS=(“walking”) OR TS=(“muscle strengthening”) |
| **#2** | TS=(“Nitric Oxide ”) OR TS=(“Nitrogen Monoxide”) OR TS=(“Nitrogen oxide”) OR TS=(“Nitric Oxide, Endothelium-Derived”) OR TS=(“Endogenous Nitrate Vasodilator”) OR TS=(“Nitrates”) OR TS=(“nitrite”) OR TS=(“Nitrosamines”) OR TS=(“Nitrogen dioxide”) |
| **#3** | TS=(Randomized controlled trial) OR TS=(controlled clinical trial) OR TS=(randomized controlled trials) OR TS=(random allocation) OR TS=(double blind method) OR TS=(single blind method) OR TS=(clinical trial) OR TS=(clinical trials) OR TS=(placebos) OR TS=(placebo) OR TS=(random) |
| **#4** | **#1** AND **#2** AND **#3** |

**SCOPUS**

|  | **Descriptors** |
| --- | --- |
| **#1** | *( ( TITLE-ABS-KEY ( exercise ) ) OR ( TITLE-ABS-KEY ( physical AND activity ) ) OR ( TITLE-ABS-KEY ( training, AND exercise ) ) OR ( TITLE-ABS-KEY ( sport ) ) OR ( TITLE-ABS-KEY ( aerobic AND training ) ) OR ( TITLE-ABS-KEY ( anaerobic AND training ) ) OR ( TITLE-ABS-KEY ( endurance AND training ) ) OR ( TITLE-ABS-KEY ( resistance AND training ) ) OR ( TITLE-ABS-KEY ( interval AND training ) ) ) OR ( TITLE-ABS-KEY ( walking ) ) OR ( TITLE-ABS-KEY ( muscle AND strengthening ) )* |
| **#2** | *( ( TITLE-ABS-KEY ( nitric AND oxide ) ) OR ( TITLE-ABS-KEY ( nitrogen AND monoxide ) ) OR ( TITLE-ABS-KEY ( nitrogen AND oxide ) ) OR ( TITLE-ABS-KEY ( nitric AND oxide, AND endothelium-derived ) ) OR ( TITLE-ABS-KEY ( endogenous AND nitrate AND vasodilator ) ) OR ( TITLE-ABS-KEY ( nitrates ) ) OR ( TITLE-ABS-KEY ( nitrite ) ) ) OR ( TITLE-ABS-KEY ( nitrosamines ) ) OR ( TITLE-ABS-KEY ( nitrogen AND dioxide ) )* |
| **#3** | ( TITLE-ABS-KEY ( randomized AND controlled AND trial ) OR TITLE-ABS-KEY ( controlled AND clinical AND trial ) OR TITLE-ABS-KEY ( randomized AND controlled AND trials ) OR TITLE-ABS-KEY ( random AND allocation ) OR TITLE-ABS-KEY ( double AND blind AND method ) OR TITLE-ABS-KEY ( single AND blind AND method ) OR TITLE-ABS-KEY ( clinical AND trial ) OR TITLE-ABS-KEY ( clinical AND trials ) OR TITLE-ABS-KEY ( placebos ) OR TITLE-ABS-KEY ( placebo ) OR TITLE-ABS-KEY ( random ) ) |
| **#4** | **#1** AND **#2** AND **#3** |

**COCHRANE**

|  | **Descriptors** |
| --- | --- |
| **#1** | Me ("Exercise") or ("Physical Activity"):ti,ab,kw or ("Training, Exercise"):ti,ab,kw or ("Sport"):ti,ab,kw or ("aerobic training"):ti,ab,kw or ("anaerobic training"):ti,ab,kw or ("endurance training"):ti,ab,kw or ("resistance training"):ti,ab,kw or ("interval training"):ti,ab,kw or ("walking"):ti,ab,kw or ("muscle strengthening"):ti,ab,kw |
| **#2** | Me ("Nitric Oxide") or ("Nitrogen Monoxide"):ti,ab,kw or ("Nitrogen oxide"):ti,ab,kw or ("Nitric Oxide, Endothelium-Derived"):ti,ab,kw or ("Endogenous Nitrate Vasodilator"):ti,ab,kw or ("Nitrates"):ti,ab,kw or ("nitrite"):ti,ab,kw or ("Nitrosamines"):ti,ab,kw or ("Nitrogen dioxide"):ti,ab,kw |
| **#3** | **#1** AND **#2** |

**EMBASE**

|  | **Descriptors** |
| --- | --- |
| **#1** | 'exercise'/exp OR exercise OR (physical AND ('activity'/exp OR activity)) OR (training, AND ('exercise'/exp OR exercise)) OR 'sport'/exp OR sport OR (aerobic AND ('training'/exp OR training)) OR (anaerobic AND ('training'/exp OR training)) OR (('endurance'/exp OR endurance) AND ('training'/exp OR training)) OR (('resistance'/exp OR resistance) AND ('training'/exp OR training)) OR (interval AND ('training'/exp OR training)) OR 'walking'/exp OR walking OR (('muscle'/exp OR muscle) AND strengthening) |
| **#2** | nitric AND ('oxide'/exp OR oxide) OR (('nitrogen'/exp OR nitrogen) AND monoxide) OR (('nitrogen'/exp OR nitrogen) AND ('oxide'/exp OR oxide)) OR (nitric AND oxide, AND 'endothelium derived') OR (endogenous AND ('nitrate'/exp OR nitrate) AND ('vasodilator'/exp OR vasodilator)) OR 'nitrates'/exp OR nitrates OR 'nitrite'/exp OR nitrite OR 'nitrosamines'/exp OR nitrosamines OR (('nitrogen'/exp OR nitrogen) AND ('dioxide'/exp OR dioxide)) |
| **#3** | (randomized AND controlled AND ('trial'/exp OR trial)) OR (controlled AND ('clinical'/exp OR clinical) AND ('trial'/exp OR trial)) OR (randomized AND controlled AND trials) OR (random AND allocation) OR (double AND ('blind'/exp OR blind) AND ('method'/exp OR method)) OR (single AND ('blind'/exp OR blind) AND ('method'/exp OR method)) OR (('clinical'/exp OR clinical) AND ('trial'/exp OR trial)) OR (('clinical'/exp OR clinical) AND trials) OR ('placebos'/exp OR placebos) OR ('placebo'/exp OR placebo) OR (random) |
| **#4** | **#1** AND **#2** AND **#3** |

***Appendix 2: Assessment of the risk of bias in the included studies***

| **Author, year (reference)** | **Random sequence generation (selection bias)** | **Allocation concealment (selection bias)** | **Blinding of participants and personnel (performance bias)** | **Blinding of outcome assessment (detection bias)** | **Incomplete outcome data (attrition bias)** | **Selective reporting (reporting bias)** | **Other bias** |
| --- | --- | --- | --- | --- | --- | --- | --- |
| Narin et al., 2013 (19) | **+** | **+** | **+** | **-** | **-** | **-** | **-** |
| Izadi et al., 2013 (16) | **-** | **+** | **+** | **?** | **+** | **-** | **-** |
| Ghahramani-Moghadam et al, 2015 (15) | **-** | **+** | **+** | **?** | **-** | **-** | **-** |
| Mohammadi et al., 2018 (18) | **-** | **+** | **+** | **?** | **-** | **-** | **-** |
| Arefirad et al., 2019 (29) | **-** | **+** | **+** | **-** | **-** | **-** | **-** |
| Hasegawa et al., 2018 (20) | **-** | **+** | **+** | **?** | **-** | **-** | **-** |
| Higashi et al., 1999 (21) | **-** | **+** | **+** | **-** | **-** | **-** | **-** |
| Maeda et al., 2004 (23) | **-** | **+** | **+** | **?** | **-** | **-** | **-** |
| Ghardashi Afousi et al., 2016 (27) | **-** | **+** | **+** | **?** | **-** | **-** | **-** |
| Tomeleri et al., 2016 (24) | **-** | **+** | **+** | **?** | **-** | **-** | **-** |
| Krause et al., 2013 (17) | **-** | **+** | **+** | **?** | **-** | **-** | **-** |
| Mourot et al., 2009 (26) | **-** | **+** | **+** | **?** | **-** | **-** | **-** |
| Wang et al., 2004 (25) | **+** | **+** | **+** | **?** | **-** | **-** | **-** |
| Tsukiyama et al., 2017 | **+** | **+** | **+** | **?** | **-** | **-** | **-** |
| Elsisi et al., 2016 (28) | - | + | + | **?** | **-** | **-** | **-** |

**+: High risk, -: Low risk, ?: Unclear**
